# Identification of a novel m6A-related lncRNAs signature and immunotherapeutic drug sensitivity in pancreatic adenocarcinoma

**DOI:** 10.1186/s12885-024-11885-8

**Published:** 2024-01-23

**Authors:** Xia-Qing Li, Shi-Qi Yin, Lin Chen, Aziguli Tulamaiti, Shu-Yu Xiao, Xue-Li Zhang, Lei Shi, Xiao-Cao Miao, Yan Yang, Xin Xing

**Affiliations:** 1 Anhui University of Science and Technology Affiliated Fengxian Hospital, 6600 Nanfeng Road, Shanghai, China; 2 Shanghai University of Medicine and Health Sciences Affiliated Sixth People’s Hospital South Campus, Shanghai, China; 3 State Key Laboratory of Systems Medicine for Cancer, Ren Ji Hospital, School of Medicine, Shanghai Cancer Institute, Shanghai Jiao Tong University, Shanghai, China; 4 School of Public Health, Lanzhou University, Lanzhou, China

**Keywords:** m6A-related lncRNAs, PDAC, Immunotherapy, Prognostic values

## Abstract

**Background:**

Pancreatic adenocarcinoma (PDAC) ranks as the fourth leading cause for cancer-related deaths worldwide. N6-methyladenosine (m6A) and long non-coding RNAs (lncRNAs) are closely related with poor prognosis and immunotherapeutic effect in PDAC. The aim of this study is to construct and validate a m6A-related lncRNAs signature and assess immunotherapeutic drug sensitivity in PDAC.

**Methods:**

RNA-seq data for 178 cases of PDAC patients and 167 cases of normal pancreatic tissue were obtained from TCGA and GTEx databases, respectively. A set of 21 m6A-related genes were downloaded based on the previous report. Co-expression network was conducted to identify m6A-related lncRNAs in PDAC. Cox analyses and least absolute shrinkage and selection operator (Lasso) regression model were used to construct a risk prognosis model. The relationship between signature genes and immune function was explored by single-sample GSEA (ssGSEA). The tumor immune dysfunction and exclusion (TIDE) score and tumor mutation burden (TMB) were utilized to evaluate the response to immunotherapy. Furthermore, the expression levels of 4 m6A-related lncRNAs on PDAC cell lines were measured by the quantitative real-time PCR (qPCR). The drug sensitivity between the high- and low-risk groups was validated using PDAC cell lines by Cell-Counting Kit 8 (CCK8).

**Results:**

The risk prognosis model was successfully constructed based on 4 m6A-related lncRNAs, and PDAC patients were divided into the high- and low-risk groups. The overall survival (OS) of the high-risk groups was more unfavorable compared with the low-risk groups. Receiver operating characteristic (ROC) curves demonstrated that the risk prognosis model reasonably predicted the 2-, 3- and 5-year OS of PDAC patients. qPCR analysis confirmed the decreased expression levels of 4 m6A-related lncRNAs in PDAC cells compared to the normal pancreatic cells. Furthermore, CCK8 assay revealed that Phenformin exhibited higher sensitivity in the high-risk groups, while Pyrimethamine exhibited higher sensitivity in the low-risk groups.

**Conclusion:**

The prognosis of patients with PDAC were well predicted in the risk prognosis model based on m6A-related lncRNAs, and selected immunotherapy drugs have potential values for the treatment of pancreatic cancer.

**Supplementary Information:**

The online version contains supplementary material available at 10.1186/s12885-024-11885-8.

## Introduction

Pancreatic ductal adenocarcinoma (PDAC) is one of the most prevalent gastrointestinal malignancies in the world and a lethal disease with extremely devastating cancer [1]. It is reported that an overwhelming number of patients with extremely aggressive PDAC in terms of 5-year survival rate only account for 6% in the United State [2]. However, the traditional treatment fails to effectively prevent the worsening tumor and reduce the mortality rate of patients with PDAC [3]. Therefore, it is necessary to develop a novel strategy to impede the rapid progression of PDAC.

Long non-coding RNAs (lncRNAs) do not have the ability of coding protein, which account for roughly four fifths in the entire transcriptome [4]. It has been demonstrated that multiple biological processes are regulated by numerous lncRNAs involved in tumor cell proliferation, apoptosis, invasion [5]. Wang et al. identified that significantly up-regulated LINC00240 were positively correlated with the poor prognosis, and silencing of the LINC00240 exhibited the decreased ability of proliferation and migration in gastric cancer [6]. Another study demonstrated that the upregulated lncRNA JPX promoted lung cancer proliferation and metastasis by the JPX/miR-33a-5p/Twist1 axis activating Wnt/β-catenin signaling both in vitro and in vivo [7]. Zhao et al. reported that the lncRNA CERS6-AS1 facilitates proliferation, migration and invasion in colorectal cancer cells by affecting miR-15b-5p to upregulate SPTBN [8].

Epigenetic modification, gene sequence is scarcely influenced, is characterized by the changed gene expression derived from various modifications, including histone modification, DNA methylation, the modification of non-coding RNA [9–11]. Particularly, N6-methyladenosine (m6A) commonly participates in the variety of biological processes. The modification of m6A functionally regulates the carcinogenesis by the three main proteins, such as binding protein (reader), demethylase (eraser), and methyltransferase (writer) [12]. The increasing research have indicated that abnormal gene expression is caused by the modification of m6A in an incorrect manner [13]. For instance, Lin et al. demonstrated that YTHDF1, the m6A reader, facilitating the immune evasion in skin cutaneous melanoma (SKCM) [14]. Moreover, Ban et al. identified that the stability of LNCAROD was augmented in a m6A modification manner and the ability of cell proliferation and mobility was significantly enhanced with the increased LNCAROD expression in head and neck squamous cell carcinoma (HNSC) [15]. Feng et al. focused on constructing an m6A-immune-related lncRNA risk model for predicting prognosis, immune landscape and chemotherapeutic response in bladder cancer [16]. However, the relationship between m6A and lncRNAs has not been deeply explored in PDAC. Therefore, it is of vital importance to develop a new treatment strategy involving m6A modification associated with lncRNAs for improving the survival time in PDAC.

In the present study, we constructed a risk prognosis model for improving the clinical outcome of patients with PDAC, and validated the rationality of the risk prognosis model and searched for appropriate immunotherapy drugs that can effectively treat PDAC. Our research aims to explore potential molecular mechanisms and treatment strategies for PDAC.

## Materials and methods

### The data sources of patients

RNA-seq data and relevant phenotype information for 178 PDAC patients were obtained from TCGA database (https://www.cancer.gov/). Meanwhile, 167 normal pancreatic tissue RNA-seq data were downloaded from the GTEx database in the UCSC Xena database (http://xena.ucsc.edu). PDAC Patients were put into train group and test group in a 1:1 ratio. The lncRNAs were annotated from the GENCODE website (https://www.gencodegenes.org). 4 lncRNA m6A binding sites were calculated by SRAMP database (http://www.cuilab.cn/sramp/).

### Establishment of m6Arelated lncRNAs signature

21 related genes were reported from previous literature [17], including writers (METTL3, METTL14, WTAP, METTL16), readers (YTHDC1, YTHDF2, YTHDC2, RBMX) and erasers (ALKBH5, FTO). The m6A-related lncRNA prognostic signature genes were designed in three steps. (1) Univariate Cox regression analysis identified those m6A-related lncRNAs significantly influencing the overall survival (OS) of PADC patients. (2) To reduce the potential for overfitting, least absolute shrinkage and selection operator (LASSO) Cox regression was applied—where the penalty parameter was calculated by performing tenfold cross-validation on the training set at the minimum partial likelihood deviance. Lastly, a multivariate Cox regression analysis was conducted to finalize the selection of ideal m6A-related lncRNAs for the prognostic signature. (3) lncRNA expression’s multivariate regression coefficients were utilized to create a prognostic signature for patients. The risk score was computed using the formula: Risk score = β1 * Exp1 + β2 * Exp2 + βn * Expn [18]. Here, ‘β’ signifies the coefficient while ‘Exp’ symbolizes the expression value of the respective m6A-related lncRNA. Patients were categorized into high- and low-risk groups based on the median risk scores. Differences in survival between these groups were illustrated using Kaplan-Meier analysis. Predictive abilities of the signature were assessed using the receiver operating characteristic (ROC) curves via the “SurvivalROC” R package. The optimal cut off point for the risk score, distinguishing patients from high- to low-risk groups, was determined using the previously described formula in the training set. The survival differences between the two groups were depicted using Kaplan-Meier analysis, and the prognostic signature’s predictive efficacy was evaluated by the time-dependent ROC curve.

### Clinical correlation analysis

Univariate and multifactorial COX regression analyses were performed to exclude the influence of other clinical factors (gender, age, TNM stage and so on) and determine independent elements about prognosis [19].

### Principal component analysis (PCA) and Kaplan-Meier survival analysis

We used Principal Component Analysis (PCA) for effective dimensionality reduction, model identification, and grouping visualization of high-dimensional data, including 21 m6A genes, 4 m6A-related long non-coding RNAs (lncRNAs), and a risk model based on the expression patterns of these 4 m6A-related lncRNAs. We employed Kaplan-Meier survival analysis to assess the differences in overall survival (OS) between high-risk and low-risk groups. The R packages were used for this process including survMiner and survival [20].

### Assessment of immune function scores and functional analysis

In our study, the single-sample Gene Set Enrichment Analysis (ssGSEA) algorithm, implemented through the R package ‘GSVA’, was utilized to evaluate differences in immune function scores between the high and low risk groups. Additionally, the Tumor Immune Dysfunction and Exclusion (TIDE) algorithm was employed to predict the efficacy of immunotherapy in these groups [21]. For differential analysis, we analyzed gene expression data from the high and low risk groups using the ‘limma’ package, adhering to specific criteria (log fold change (log FC) > 1 and a false discovery rate (fdr) < 0.05). Based on the findings of this differential analysis, Gene Ontology (GO) enrichment analysis was conducted using the ‘clusterProfiler’ package to identify and characterize significant GO terms associated with the differentially expressed genes, thereby providing insights into the biological processes and pathways that may underlie the risk stratification observed in our study [19].

### Comparison of the tumor mutation burden (TMB)

The “maftools” R package’s waterfall function was employed to illustrate the mutation panorama in both the high-risk and low-risk groups [22]. This functionality allowed us to quantify the somatic mutation count and TMB, expressed as mutations per million bases, for each individual patient. To discern any statistical differences in the somatic mutation count and TMB levels between the high- and low-risk groups, Wilcoxon test was conducted. The same “maftools” R package was utilized to determine TMB within the high and low-risk groups. Subsequent to this, we employed the Kaplan-Meier methodology to compare survival rates. This allowed us to assess the differences between the high and low mutation groups, and the risk categories. This varied approach offered a comprehensive view of the mutation landscape in correlation with patient risk level and survival rates.

### Drug sensitivity

The R package “pRRophetic” was utilized to forecast the IC50 of drugs deriving from the GDSC (https://www.cancerrxgene.org/) website site summary in patients with PDAC.

### Cell culture

Human pancreatic adenocarcinoma cells (AsPC-1, PANC-1, MIA PaCa-2, Patu8988, CFPAC-1, SW1990, BxPC-3), and human normal pancreatic duct epithelial cell (HPDE) were all preserved in Shanghai Cancer Institute, Shanghai Jiao Tong University. AsPC-1, BxPC-3, CFPAC-1 were kept in RPMI 1640 with 10% fetal bovine serum (FBS), and or PANC-1, Patu8988, MIA PaCa-2, SW1990, HPDE were cultured by DMEM with 10% FBS. The ratio of cell passage is subtlety different according to the cell growth rate, typically ranging from 1:2 to 1:3. Cell passaging was performed when the cell density reached 80-90% confluence in the bottom of culture dish. The old medium in the dish was discarded, and the cells were washed by PBS 1–2 times. Subsequently, the trypsin solution containing EDTA was added into the incubator (5% CO_2_ at 37 °C) with 2–5 min. To prevent further digestion, DMEM or RPMI 1640 supplemented with 10% FBS was added to the dish when the 70%~80% cells appeared round and the enlarged cell space were observed in the inverted microscope. After that, the cell suspension was transferred to a 15mL centrifuge tube and centrifuged at 800 rpm/min with 3–5 min. Next, supernatant was discarded and the new medium was used for resuspended cells. Finally, cells were inoculated into a new dish appeared round and were suspended in the indicated cell culture medium. The cells gradually returned to the original shape and sticked to the bottom of plate after 4 h. All cells were cultured under the same condition (5% CO_2_ at 37 °C).

### RNA extraction and qPCR

Appropriate Trizol (ShareBio, Shanghai, China) was added into the cells in the dish washed by PBS with 1–2 times (10 cm dish with 2mL, 6 cm dish with 1mL). Then, 1 ml cell suspension was transferred to the 1.5 mL EP tubes after placing at ice for 5 min. 200µL chloroform was added into cell suspension, mixed well for 15s and rested on ice for 3-10 min. After that, centrifugation was performed at 4℃, 12,000 rpm/min for 15 min. Next, the upper water phase was taken into a new EP tube (200–500µL), and then added by equal volume isopropyl alcohol and rested on the ice for 10 min. The cell suspension was centrifuged at 4℃, 12,000 rpm/min for 10 min, and cells were kept and washed with 500µL anhydrous ethanol at twice. The cells were dissolved in DEPC water after drying 15 min. The concentration of RNA was measured and adjusted to 500ng/µL.

The first-strand cDNA was reverse-transcribed by All-in-One First-Strand Synthesis Master Mix reagent Kit (ShareBio, Shanghai, China). The reverse transcription system was defined as follows: Template RNA: 50 ng ~ 1 µg (1µL), All-in-One First-Strand Synthesis MasterMix: 4 µL, dsDNase:1 µL, 10x dsDNase Buffer: 2 µL, Nuclease-Free Water: 12 µL. Secondly, Mix gently and spin briefly. Thirdly, incubate under 37℃ for 2 min to remove the gDNA contamination. Fourthly, Incubate under 55℃ for 15 min. Then, Terminate the reaction by incubate under 85℃ for 5 min. Finally, the cDNA was put on ice immediately and diluted by DEPC (1:20).

The 10µL system of qPCR per well was defined as follows: 0.5µL Forward primer, 0.5µL Reverse primer, 5µL SYBR green qPCR Premix (ShareBio, Shanghai, China) and 4µL cDNA. qPCR was performed with on a 7500 Real-time PCR system (Applied Biosystems, Inc. USA). The expression levels of target genes were calculated by compared to the expression of the reference gene 18s, and quantification was performed using the 2^-ΔΔct^method. The related experiments were performed in triplicate. The primers used in this study were shown in Supplementary Table 1.

### IC50

To identify the sensitivities of drugs for different cell lines, the concentration gradient (0, 1, 10, 50, 100, 200, 500, 1000µM) was defined in the Phenformin (Selleck.cn, Shanghai, China) and Pyrimethamine (Selleck.cn, Shanghai, China). The cells were suspended in 100µL DMEM with 10% FBS. 3 × 10^3^ HPDE, MIA PaCa-2 and Patu8988 were seeded into 96-well plates in the different concentration gradient and drugs treatment groups. Then, the 96-well plates were placed into the incubator (5% CO_2_ at 37 °C) for 24 h. The cells were treated by the Phenformin or Pyrimethamine with different concentration gradient (0, 1, 10, 50, 100, 200, 500, 1000µM) with 36 h. After that, Cell viability was measured by 10% CCK8 reagent (Share-Bio, shanghai, China, SB-CCK8L) at the incubator (5% CO_2_ at 37 °C) with 1 h. The absorbance of each 96-well plates was measured at 450 and 600 nm by microplate reader, and the half-maximal inhibitory concentration (IC50) was calculated by non-linear regression using GraphPad Prism 9.0.

### Cell-counting kit 8

In order to evaluate cell cytotoxicity, 3 × 10^3^ MIA PaCa-2 or Patu8988 per well in 96-well plate were plated in different groups with/without Phenformin or Pyrimethamine treatment. The cells were cultured in the incubator (5% CO_2_ at 37 °C) with 24 h. Then, the Phenformin (0 µM, 100 µM, 200 µM, 400 µM) or Pyrimethamine (0 µM, 50 µM, 100 µM, 200 µM) of different concentration gradient was added into MIA PaCa-2 and Patu8988 for 36 h, respectively. 100 µL of CCK-8 mixture (CCK-8 reagent: DMEM = 1:9) were added into these cells at the incubator (5% CO_2_ at 37℃) for 2 h. The absorbance of each well was observed at 450 and 600 nm. Finally, the optical density at 450 nm in different groups was calculated using GraphPad Prism 9.0.

### Statistical analysis

Statistical analysis was performed using R 4.1.3 software and GraphPad Prism 9.0. The wilcoxon rank-sum test and Mann-Whitney U test were used to compare the continuous variables and Spearman analysis to calculate correlation coefficients. Kaplan-Meier method was used to draw survival curve, and log-rank test was used to compare survival differences. The IC50 was calculated by Nonlin fit. *P* < 0.05 was recognized as statistically significant.

## Results

### Identification of m6A-related lncRNAs in PDAC

**Fig. 1:**
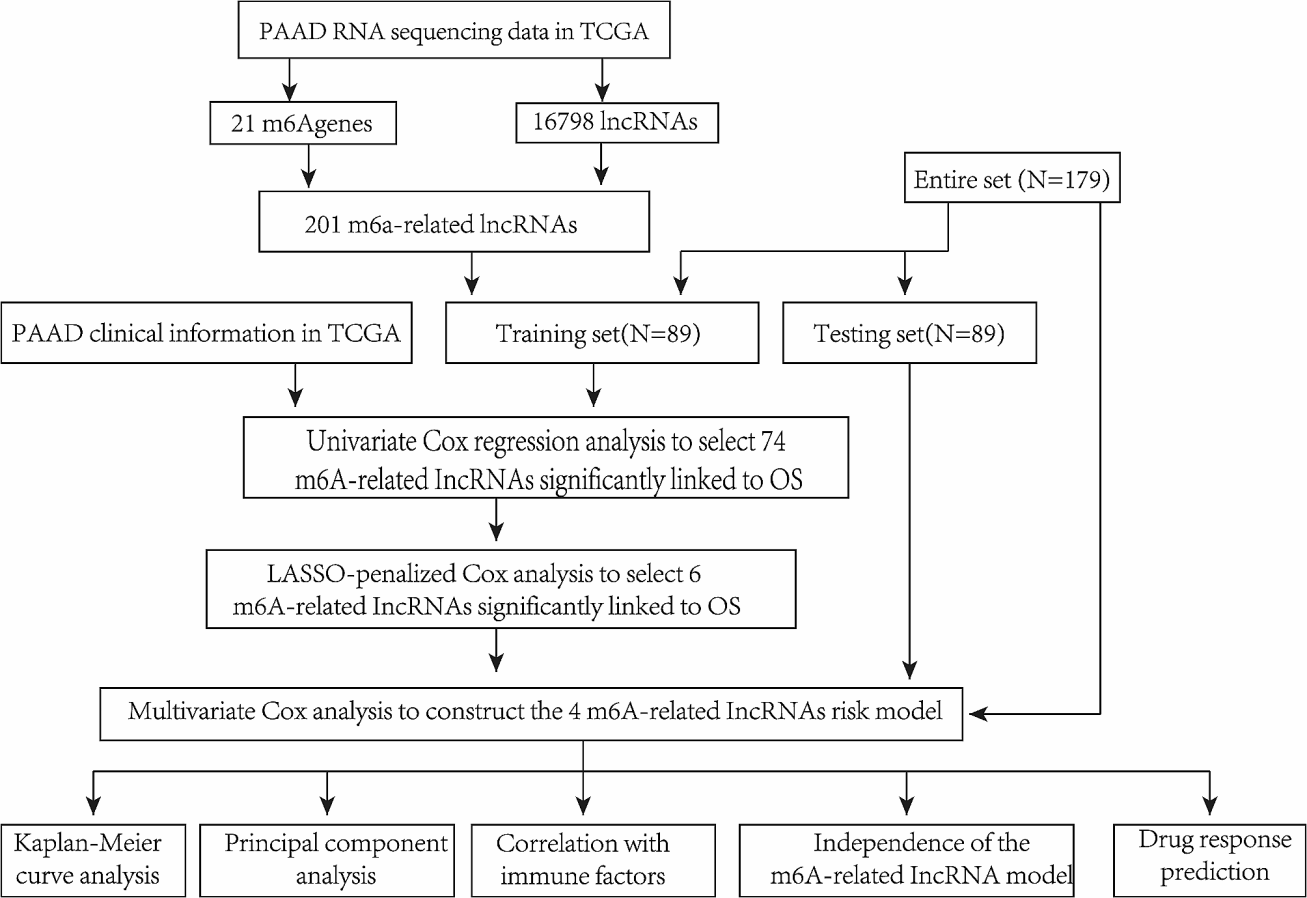
Flow chart of this study

**Fig. 2:**
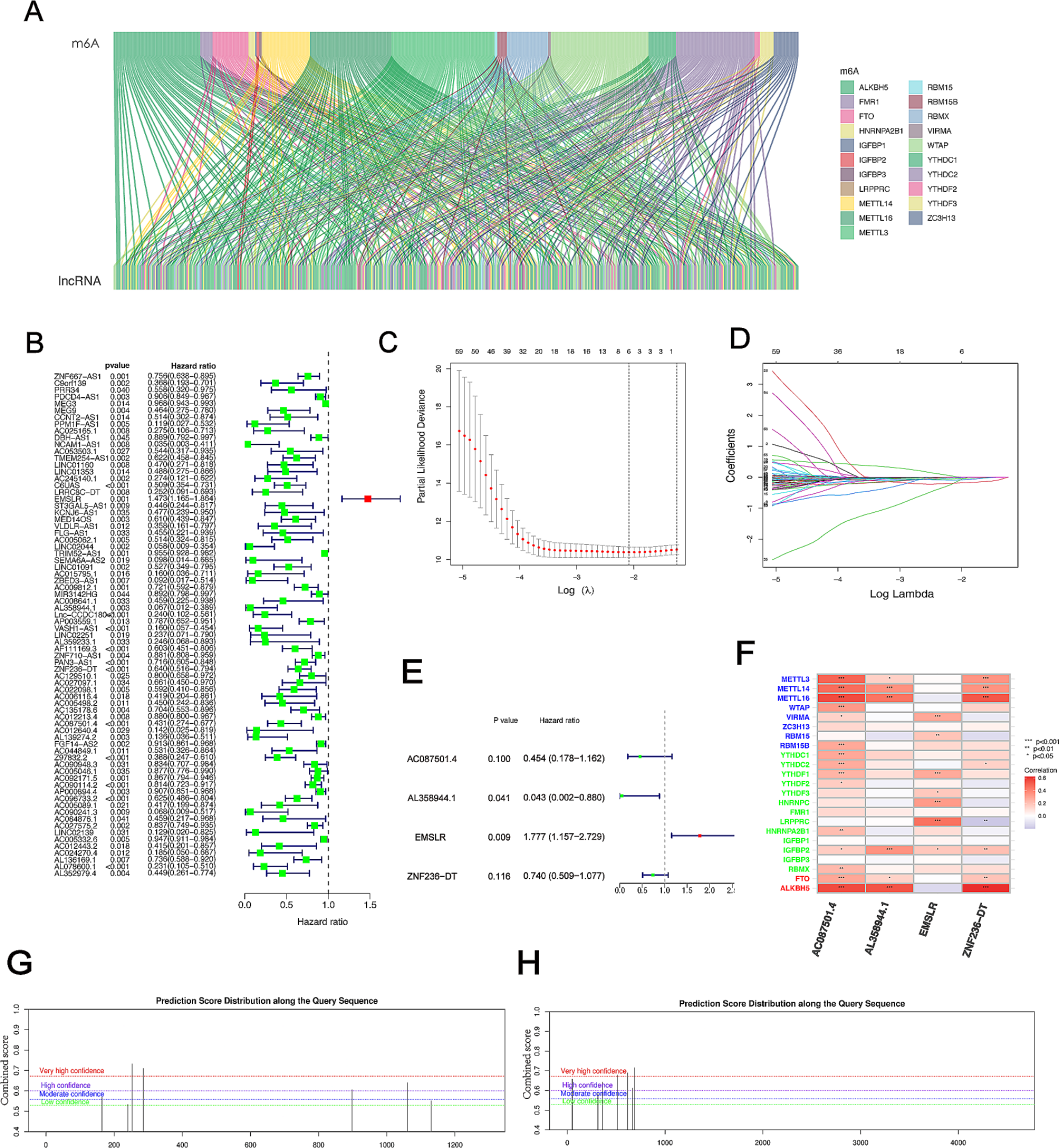
Identification of m6A-related lncRNAs in patients with PDAC. (A) Sankey diagram demonstrating co-expression relationship between 21 m6A genes and 201 m6A-related lncRNAs. (B) Univariate Cox regression analysis were applied to recognize m6A-related lncRNAs associated with prognosis. (C) Optimal prognosis model was constructed by Lasso regression analysis with tenfold cross validation. (D) Lasso coefficient curve. (E) Multivariate Cox regression analysis was utilized for m6A-related lncRNAs. (F) Heatmap displaying the correlation between 4 lncRNAs and gene associated with m6A. A sequence-based N6-methyladenosine (m6A) modification site predictor of ZNF236-DT (G) and EMSLR (H)

Using a specific workflow, we retrieved numerous gene sets from the TCGA-PAAD database and identified 16,876 lncRNAs from the total genes (Fig. 1). 21 m6A genes and 16,876 lncRNAs were matched by the utilization of gene co-expression analysis. After the above analysis, 201 m6A-related lncRNAs were successfully filtered (|Pearson R| >0.3, *P* < 0.001) (Fig. 2A). Next, a univariate Cox analysis was performed based on correlation between m6A-related lncRNAs and survival characteristics of patients with PDAC. Then, 74 m6A-related lncRNAs were filtered according to statistical analysis (Fig. 2B). To identify potential biomarkers influencing clinical outcome of patients with PDAC, we proposed a Lasso regression model, in which regression coefficients (using the penalty parameter estimated by 10-fold cross validation) were accurately calculated. 6 lncRNAs were dissociated from 74 m6A-related lncRNAs (Fig. 2C, D). Eventually, 4 m6A-related prognostic lncRNAs were yielded and validated by multivariate Cox regression analysis, including AC087501.4 (HR = 0.454, *P* = 0.1), AL358944.1 (HR = 0.043, *P* = 0.041), EMSLR (HR = 1.777, *P* = 0.009), ZNF236-DT (HR = 0.74, *P* = 0.116) (Fig. 2E and Figure S1A-D). Then, we pictured heatmap of correlation between m6A-related lncRNAs and m6A regulators and drew a significant result that there was a strong correlation between screened lncRNAs and m6A-related regulators (Fig. 2F). Finally, m6A modification sites of those 4 lncRNAs were predicted by SRAMP, which further confirmed that those 4 lncRNAs were regulated by m6A modification (Fig. 2G, H and Figure S1E, F).

### Validation of a risk model in PDAC patients

**Fig. 3:**
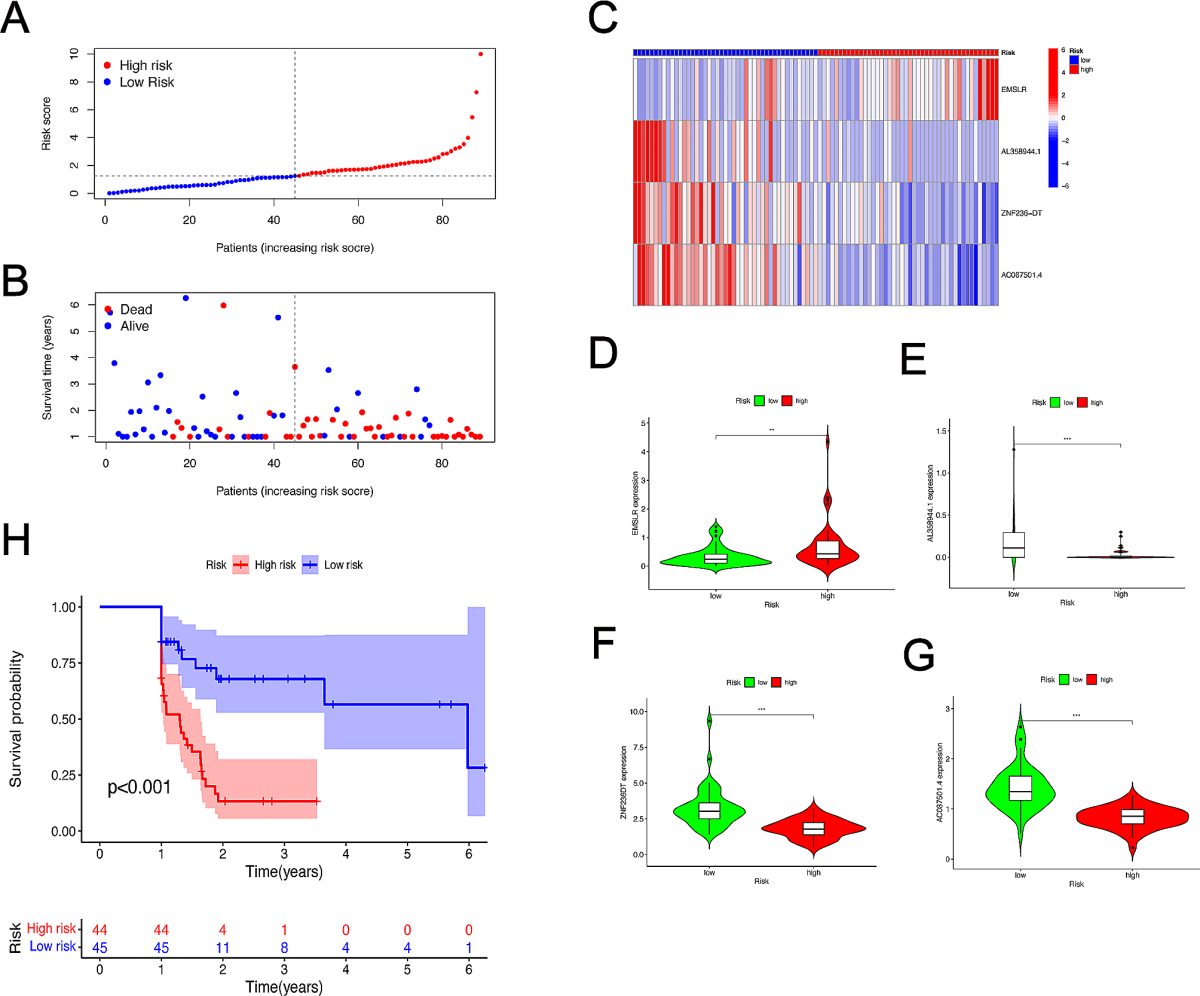
Prognostic validation of m6A-realted lncRNAs in risk model in the training sets. (A) Difference of survival status and survival time in the high- and the low-risk groups in the training sets. (B) Aggregation of m6A-realted lncRNAs based on distinct risk scores in the training sets. (C) A heat map of 4 prognostic lncRNAs related m6A gene in the training sets. The expression differences of EMSLR (D), AL358944.1 (E), ZNF236-DT (F), AC087501.4 (G) in high- and low-risk groups in the training sets. (H) Kaplan-Meier survival curves demonstrating different OS of patients with PDAC in the high- and the low-risk groups in the training sets

In order to constructed the risk prognosis model, the patients with PDAC (*n* = 178) were accurately divided into the high- and the low-risk groups according to the median cut-off point from prognostic risk scores. EMSLR was immensely higher in the high-risk groups compared with the low-risk groups. Nevertheless, the clinical information from patients with PDAC and prognostic risk scores were considered in comprehensive analysis, indicating that mortality rate of patients with PDAC was positively correlated with the risk scores (Figure S2A, B). AL358944.1, ZNF236-DT, AC087501.4 exhibited relatively low expression in the high-risk groups (Figure S2C-G). Patients with PDAC who were in high-risk groups (*n* = 89) possessed lower OS than those in low-risk groups (*n* = 89) (*p* < 0.001) (Figure S2H). To prove rationality of model, we randomly separate into a training cohort (*n* = 89) and a test cohort (*n* = 89), the increasing risk scores with worse outcome was observed in the high-risk groups (Fig. 3A, B). Besides, the highly expressed EMSLR (*p* < 0.05) and the lowly expressed AL358944.1 (*p* < 0.001), ZNF236-DT (*p* < 0.05), AC087501.4 (*p* < 0.001) were identified in the high-risk groups (Fig. 3C-G). Eventually, patients in the high-risk groups obtained poor prognosis (*p* < 0.001) (Fig. 3H). Similarly, the same tendency was shown in the test cohort, patients with high-risk scores occupied a large proportion in dead individuals (Figure S3A, B). Then, EMSLR was recognized as a risk factor and lowly expressed AL358944.1, ZNF236-DT, AC087501.4 exerted a profound function on contributing to development of cancer (Figure S3C-G). Finally, the groups of high-risk scores tend to get shorter OS than the low-risk groups (*p* < 0.001) (Figure S3H). These results suggest that the risk model is reasonably constructed and can predict the prognosis of PDAC patients.

### Comprehensive analysis of a risk model in clinical subgroup

**Fig. 4:**
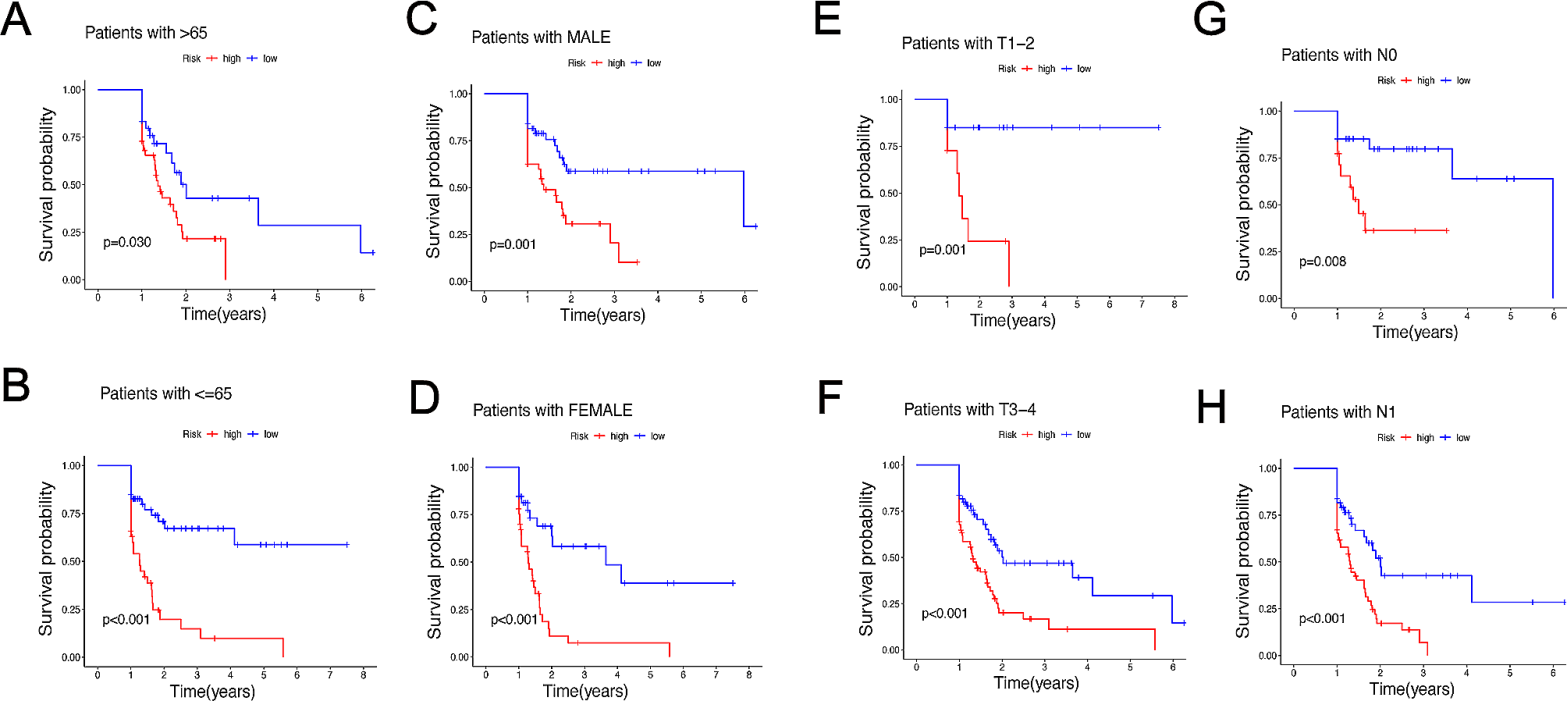
Kaplan-Meier curves of OS differences layered by age (A, B), gender (C, D), tumor grade (E, F), or TNM stage (G, H) between the high- and low-risk groups from TCGA data set

The clinical phenotype information of patients with PDAC were initially obtained from TCGA-PAAD. Then, patients with PDAC were layered by clinicopathologic characteristics, such as age (≤ 65 vs. >65), gender (male vs. female), tumor grade (G1-2, G3-4), N stage (N0, N1), T stage (T1–2, T3-4), stage (I-II, III-IV) and M stage (M0, M1). The K-M survival curve indicated that OS for the high-risk groups was significantly lower than that those in the low-risk groups no matter age over 65 (*P* = 0.03) or under 65 (*P* < 0.001) (Fig. 4A, B). Similarly, the high-risk groups possessed worse prognosis compared with the low-risk groups in both male (*P* = 0.001) and female (*P* < 0.001) patients (Fig. 4C, D). In addition, patients with the high-risk groups showed more unfavorable clinical outcome than those in the low-risk groups in T1–2 (*P* = 0.001), T3-4 (*P* < 0.001), N0 (*P* = 0.008), N1 (*P* < 0.001), stage I-II (*P* < 0.001) (Fig. 4E-H and Figure S4A). However, there is no significant difference to prognostic survival between the high- and low-risk groups in stage III-IV (*P* = 0.228) (Figure S4B), M0 (*P* = 0.073) (Figure S4C), M1 (*P* = 0.564) (Figure S4D). In brief, the prediction of clinical prognosis of patients with PDAC was greatly performed in the most subgroups.

### PCA analysis related with risk prognosis model

**Fig. 5:**
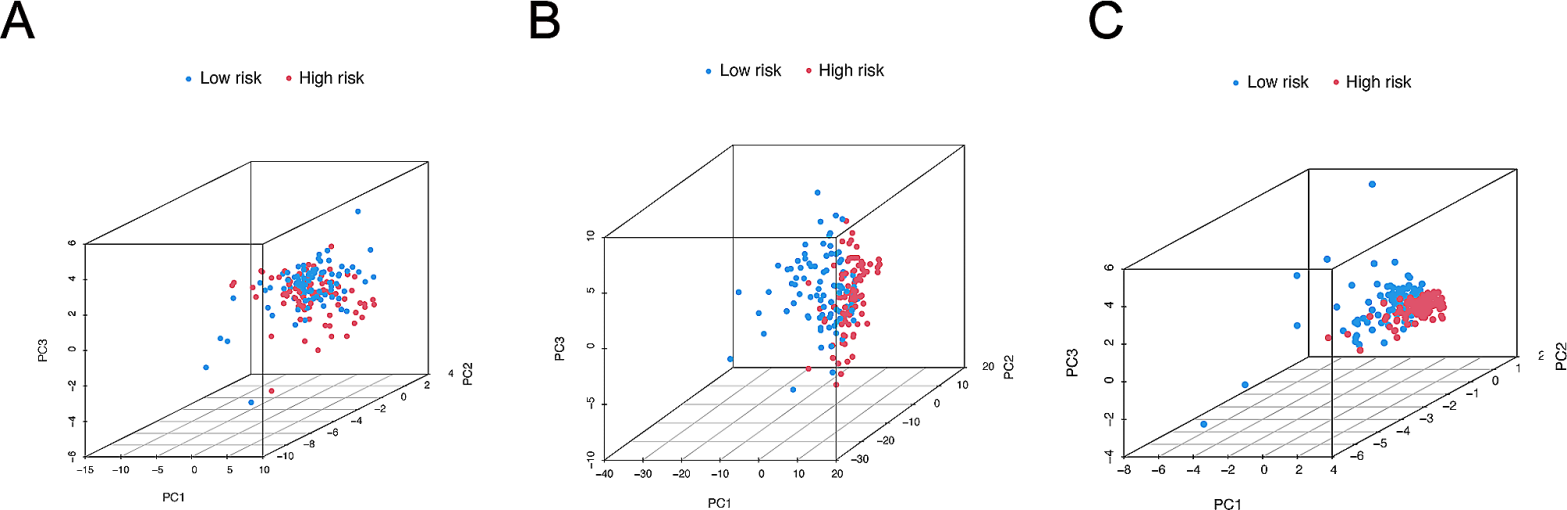
Desperation patients from TCGA-PAAD sets based on m6A-related lncRNAs. (A) PCA map of genes related to m6A. (B) Distribution of m6A-related lncRNAs using PCA map. (C) lncRNAs PCA plot according to risk scores

To investigate distribution between the high- and the low-risk groups, PCA was mainly utilized to further make distinct samples independent from each other. m6A genes, m6A-related lncRNAs and lncRNAs related with risk model were respectively evaluated. In the three-dimensional space, m6A gene and m6A-related lncRNAs between the high and the low-risk groups were mixed together (Fig. 5A-B). However, lncRNAs related with risk model were effectively distinguished in the high- and low- risk groups (Fig. 5C). Therefore, this indicates that the risk model has the capability to accurately distinguish between different expression groups and has potential clinical relevance for PDAC patients.

### Validation of influencing tumor immune therapy and tumor mutation burden

**Fig. 6:**
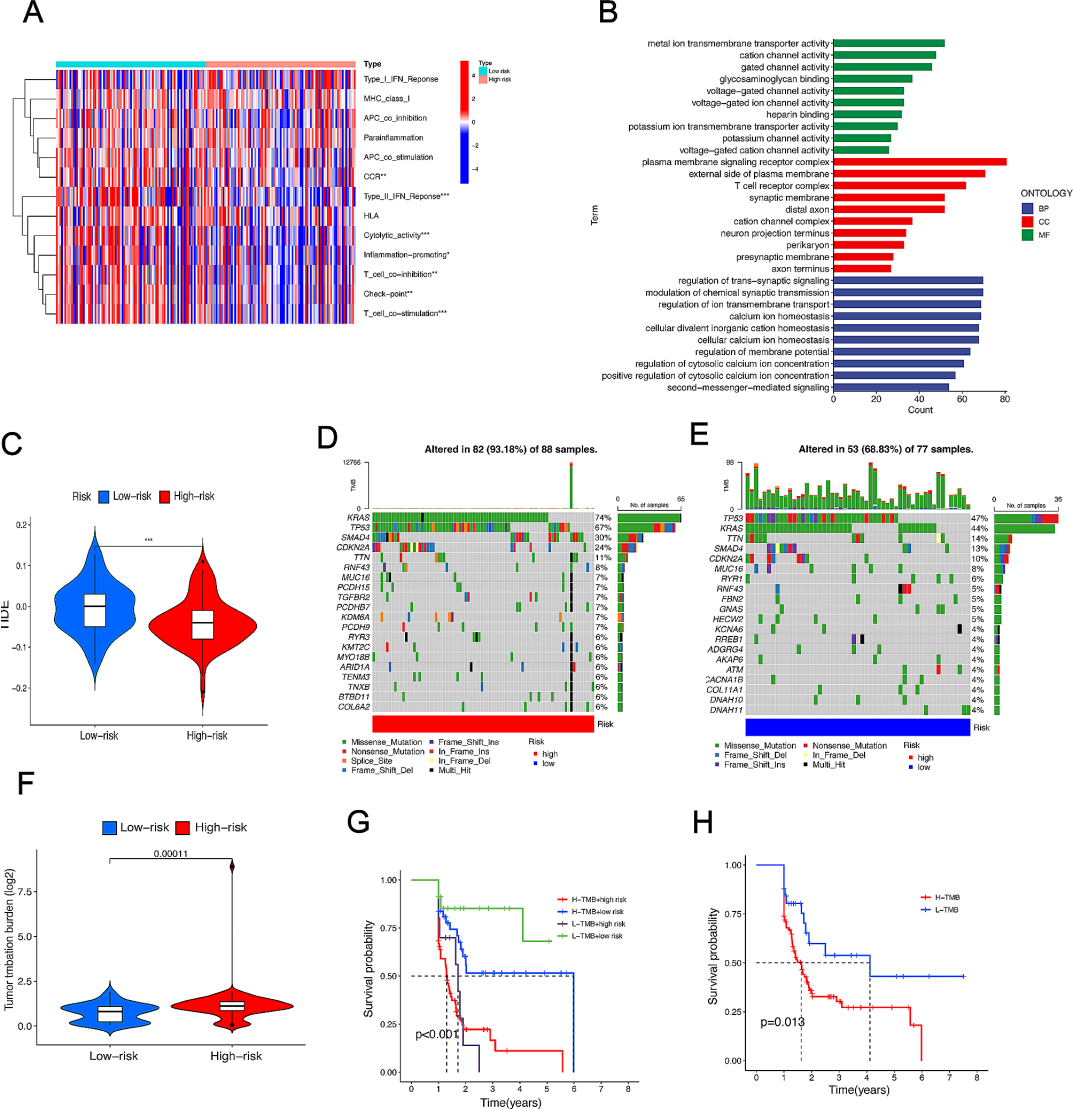
Validation of influence tumor immune therapy and tumor Mutation Burden exert using the m6A-related lncRNA model in TCGA sets. (A) A heat map indicating expression of immune molecules in the high- and the low-risk groups. (B) GO enrichment analysis. (C) TIDE differences between the high- and the low-risk groups. (D and E) Waterfall plot showing different mutation frequencies in the high-risk groups (D) and the low-risk groups (E). (F) TMB differences in the high- and the low-risk groups. (G) Kaplan-Meier curve analysis indicates relationship between prognosis of patients with PDAC and TMB level. (H) Outcome of patients with different TMB level influenced by risk scores

Currently, the increasing research have indicated that the function of immune cell is disturbed by the abnormal tumor microenvironment caused by tumor cells [23]. Consequently, enrichment analysis was performed from differential genes (DEGs) between the high- and low-risk groups. Intriguingly, immune-related cells and immune components, including Type-I-IFN-Response, MHC-class-I, APC-co-inhibitor, were extremely different at the expression level in the high- and low-risk groups (Fig. 6A). To go deeply into potential pathways the m6A-related lncRNAs predominantly participate in, ssGSEA was utilized to evaluate differences in immune function between the high- and low-risk groups. GO enrichment analysis suggested that a host of biological processes associated with immune were involved in the development of PDAC, such as T cell receptor complex (Fig. 6B). To explore sensibility of patients with PDAC accepting immune checkpoint blockade therapy, tumor immune dysfunction and exclusion (TIDE) was utilized in the low- and high-risk groups. The lower TIDE scores were obtained in the high-risk groups than the low-risk groups, which illustrated that patients in the high-risk groups were prone to gain more favorable immune checkpoint blockade (ICB) efficiency than those in the low-risk groups (Fig. 6C). In recent years, ICB has gradually become an emerging cancer immunotherapy [24, 25]. The infiltration degree of cytotoxic lymphocyte(CTL)may be immensely enhanced by combination with ICB, such as programmed death-ligand 1 (PD-L1), programmed death 1 (PD-1) and cytotoxic T lymphocyte-associated protein 4 (CTLA4) for those with the high-risk groups. In this way, the OS of patients with PDAC is expected to be further improved. To gain insight into relationship between tumorigenesis of pancreatic cancer and gene mutation, gene alteration frequency was calculated at length in the low- and high-risk groups separately. The top 20 gene mutation was shown using the R package (Fig. 6D, E). It is universally acknowledged that tumor mutation burden (TMB) is positively correlated with efficiency of tumor immunotherapy. Thus, TMB scores was counted based on Neoplastic antigen prediction software, indicating that TMB scores was higher in the high-risk groups than the low- risk groups (Fig. 6F). Kaplan-Meier demonstrated that high-TMB patients with PDAC obtained poor overall survival. Simultaneously, OS of patients with high-TMB was drastically worse in high-risk groups (Fig. 6G, H). Briefly, these findings show that the m6A-related lncRNA model play an indispensable role in predicting prognostic value of patients with PDAC.

### Estimation of the relationship between the risk model and clinical characteristics

**Fig. 7:**
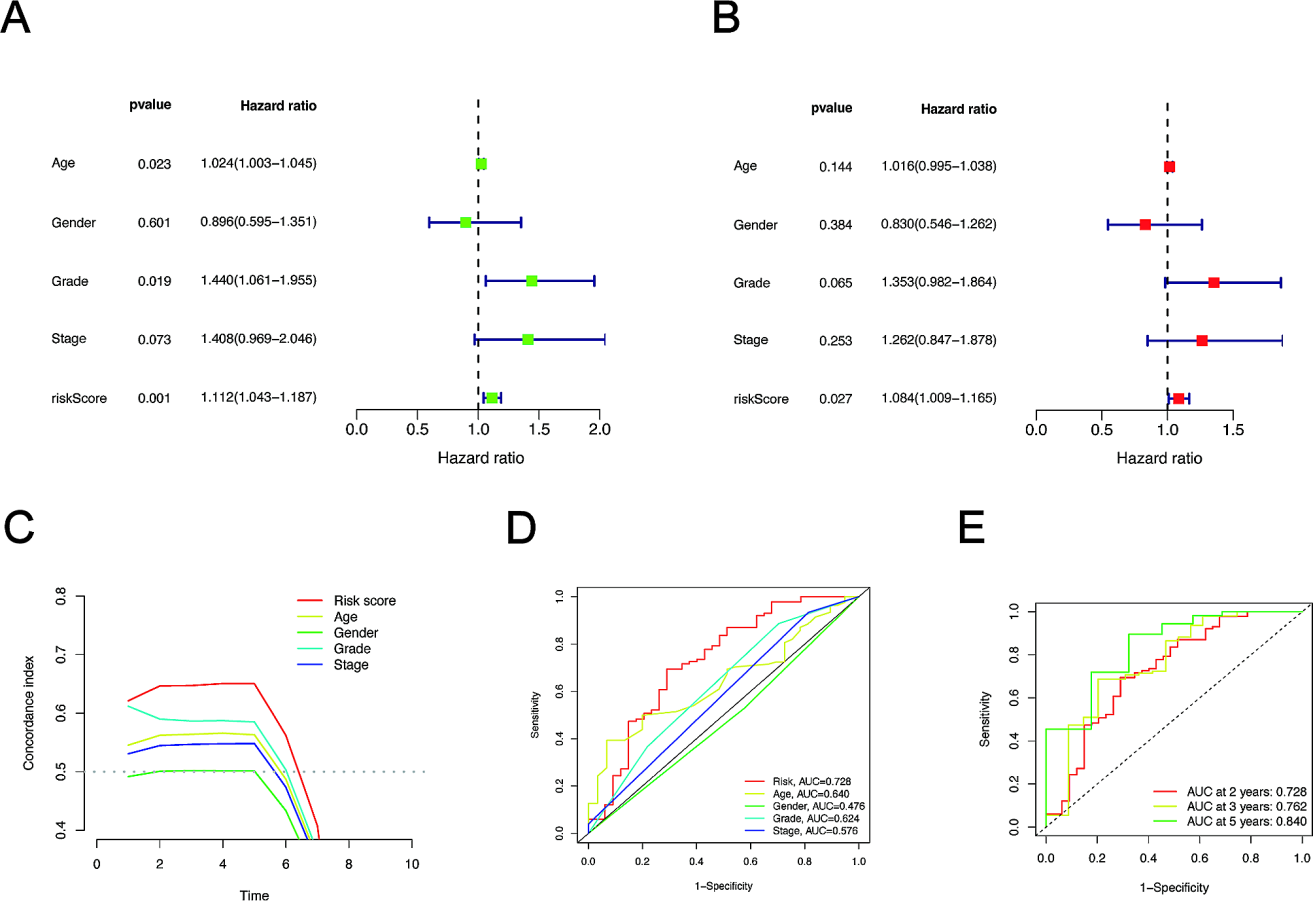
Estimation of the relationship between the risk model of m6A-related lncRNAs and clinical characteristics in TCGA data. Univariate (A) and multivariate (B) Cox regression analyses were utilized to assess OS of patients influenced by clinical characteristics and risk score. (C) Concordance indexes of the risk score and clinical characteristics. (D) ROC curves of the clinical parameters and risk score. (E) ROC analysis for predicting the 2, 3 and 5year OS.

To explore whether m6A-related lncRNAs in the risk model were significantly correlated with prognosis of patients with PDAC, univariate Cox regression analyses was performed. In univariate Cox regression model, The HR of the risk score and 95% confidence interval (CI) were 1.112 and 1.043–1.187 (*p* = 0.001) separately. Thus, the risk scores in m6A-related lncRNAs are considered as an independent prognostic risk factor (Fig. 7A). Then, multivariate Cox analysis, including risk scores and other Clinicopathological parameters, further demonstrated that the risk scores are the only independent prognostic characteristics for PDAC (Fig. 7B). The conformance index of the risk scores was seriously calculated to evaluate specificity and sensitivity of risk scores in predicting prognostic value with PDAC patients. These results indicated that the concordance index of the risk score was higher compared with other clinical parameters (Fig. 7C). Similarly, the area under the ROC curve (AUC) was larger in the risk grade (AUC = 0.728) than other clinical characteristics, including age (AUC = 0.640), gender (AUC = 0.476), grade (AUC = 0.0.624), stage (AUC = 0.0.576), indicating that the risk model of m6A-related lncRNAs applied for predicting prognosis of patients with PDAC is greatly reliable (Fig. 7D). Finally, ROC curve was performed to predict the 2 (AUC = 0.728), 3 (AUC = 0.0.762) and 5 (AUC = 0.840) year OS with patients of PDAC, indicating that the 4 m6A-lncRNAs have good prognostic value (Fig. 7E).

### Identification of sensitive drugs in PDAC patients

**Fig. 8:**
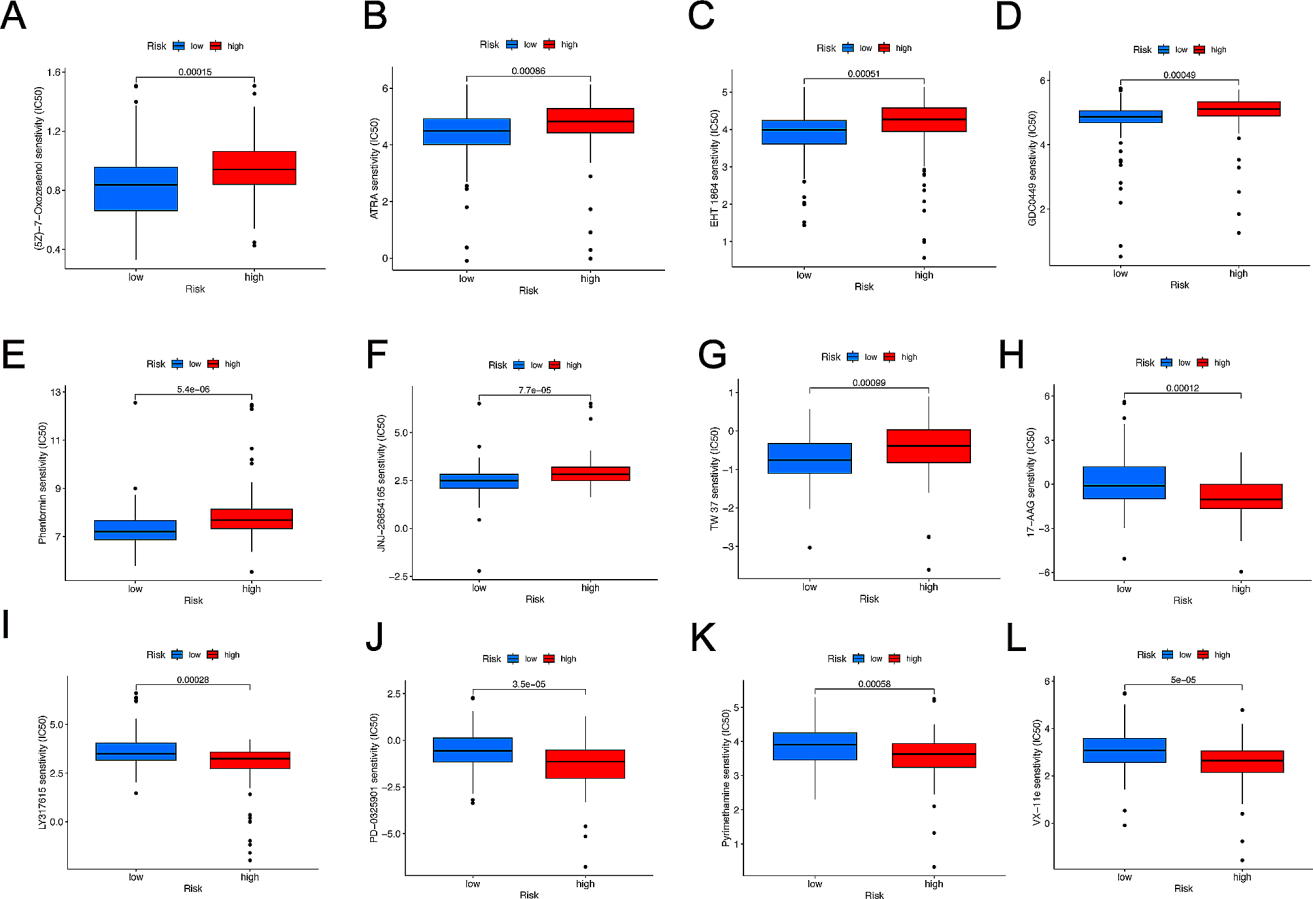
IC50 of different immunotherapy drugs according to distinct risk score. (A) (5Z)-7-Oxozeaenol, (B) ATRA, (C) EHT 1864, (D) GDC0449, (E) Phenformin, (F) JNJ-26,854,165, (G) TW 37, (H) 17-AAG were more sensitive in the high-risk groups. (I) LY317615, (J) PD-0325901, (K) Pyrimethamine, (L) VX-11e were more sensitive in the low-risk groups

To recognize effective immunotherapy drugs for patients with PDAC, 12 immunotherapy drugs were identified. (5Z)-7-Oxozeaenol (*p* = 0.00015), ATRA (*p* = 0.00086), EHT 1864 (*p* = 0.00051), GDC0449 (*p* = 0.00049), JNJ-26,854,165 (*p* = 0.000077), Phenformin (*p* = 0.0000054), TW 37 (*p* = 0.00099) were higher in the high-risk groups as targeted treatment drugs (Fig. 8A-G). However, in the high-risk groups, 17-AAG (*p* = 0.00012), LY317615 (*p* = 0.00028), PD-0325901 (*p* = 0.000035), Pyrimethamine (*p* = 0.00058), VX-11e (*p* = 0.00005) were discovered in low level (Fig. 8H-L). Therefore, distinct immunotherapy drugs in suitable doses were used for different patients with PDAC based on the risk scores.

### Different sensitive immunotherapy drugs in the high- and the low-risk groups inhibited proliferation of PDAC cells

**Fig. 9:**
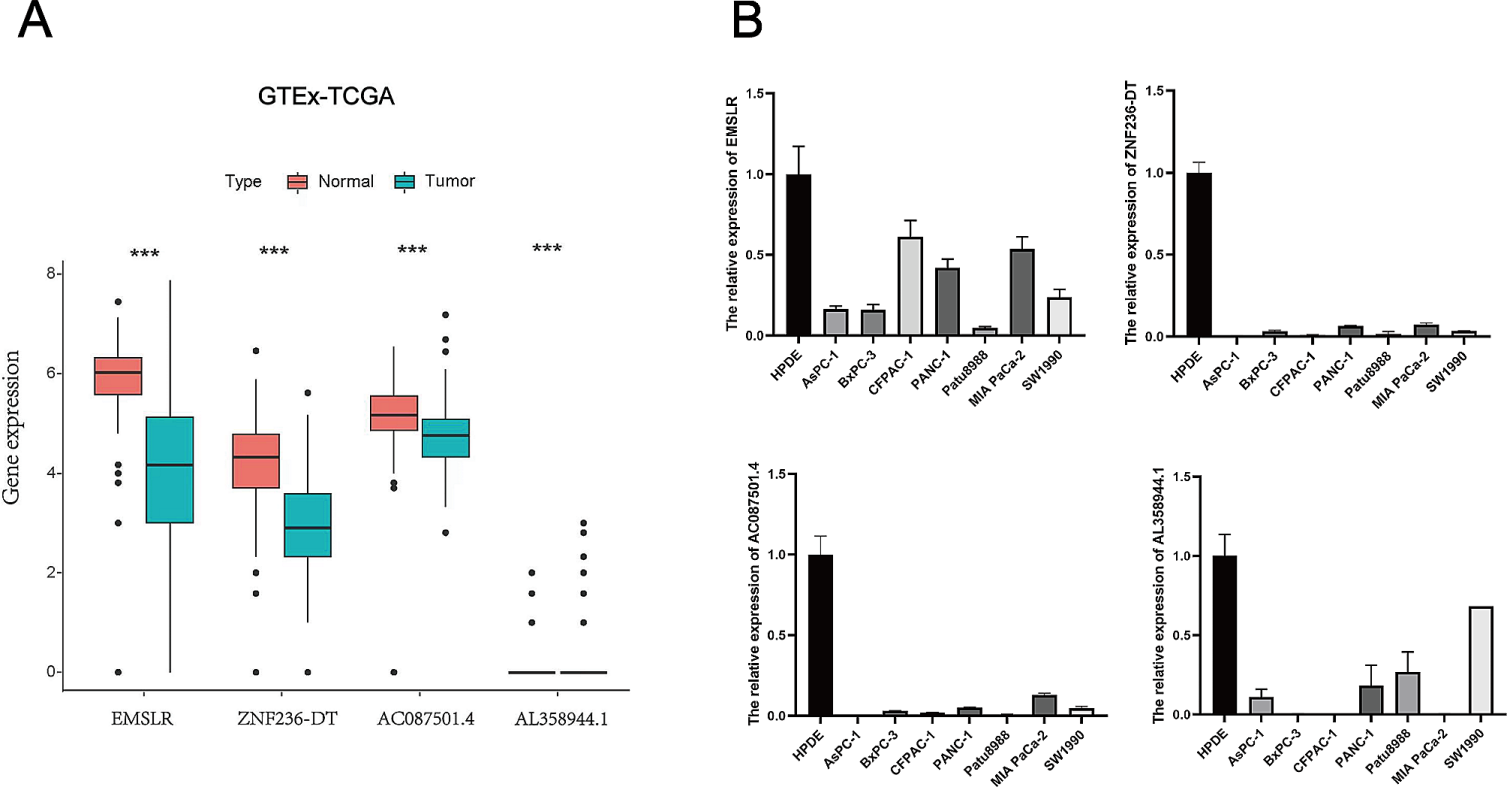
The expression levels of m6A-related LncRNAs in PDAC. (A)The boxplot showing that differential expression of EMSLR, ZNF236-DT, AC087501.4, AL358944.1 in PDAC using TCGA-GTEx. (B) The mRNA expression levels of EMSLR, ZNF236-DT, AC087501.4, AL358944.1 were detected by qPCR in PDAC cell lines

**Fig. 10:**
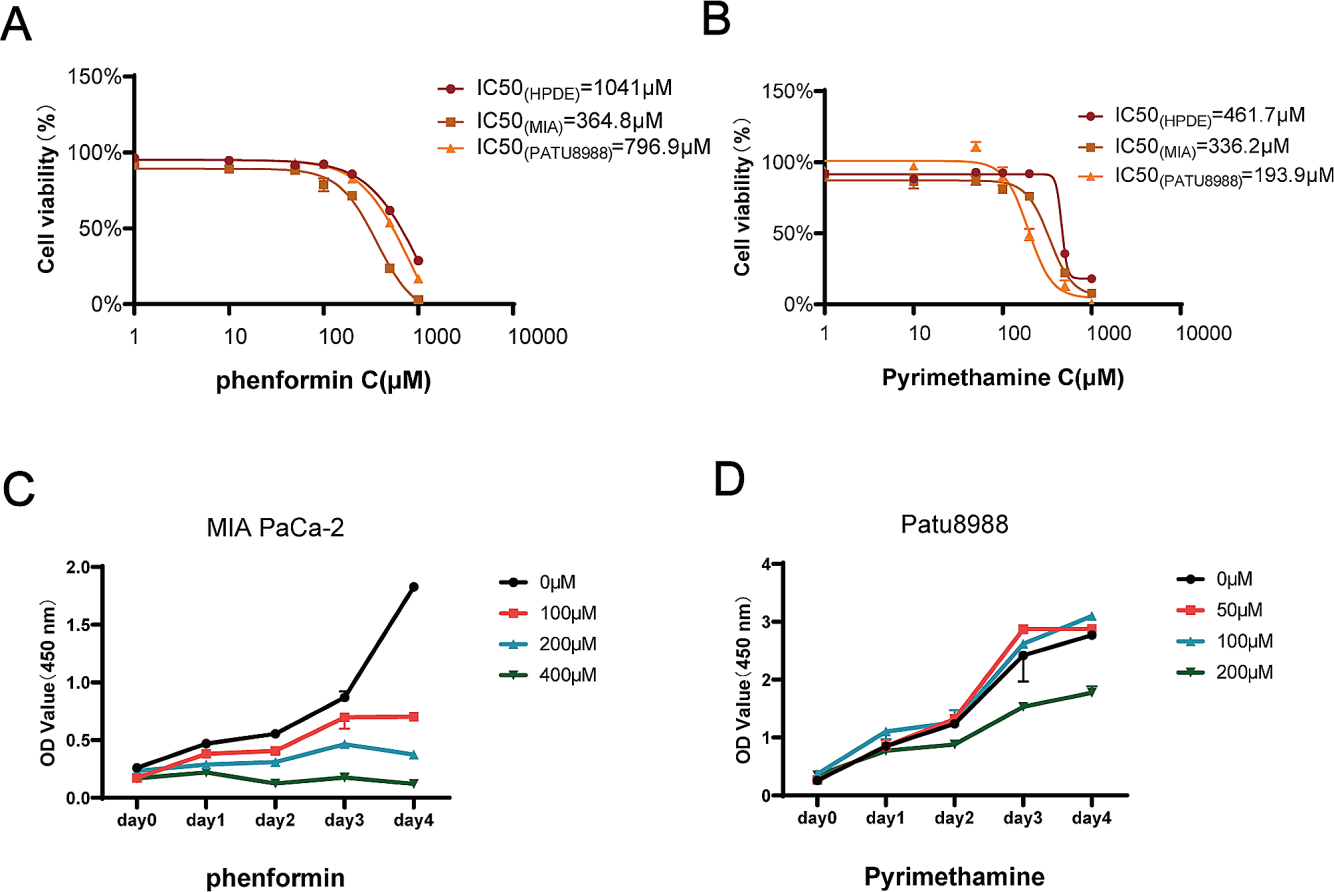
MIA PaCa-2 and Patu8988, which were treated with Phenformin and Pyrimethamine respectively, displayed slow proliferation. IC50 was evaluated by CCK8 in HPDE, MIA PaCa-2 and Patu8988 cells treated with Phenformin (A) and Pyrimethamine (B) Cell proliferation was measured by CCK8 assay in MIA PaCa-2 treated with Phenformin (C) and Patu8988 treated with Pyrimethamine (D) at different doses for 4 days

The expression level of EMSLR, ZNF236-DT, AC087501.4, AL358944.1 were lower than normal group by TCGA-GTEx (Fig. 9A). Furthermore, qPCR results showed that the four lncRNAs were significantly decreased in MIA PaCa-2, AsPC-1, PANC-1, CFPAC-1, BxPC-3, Patu8988, SW1990 cells compared with normal pancreatic HPDE cells (The degree of decline in different PDAC cell lines: AL358944.1 (31.67-99.87%), EMSLR (38.86-95.37%), ZNF236-DT (92.71-99.46%), AC087501.4 (87.17-99.45%)) (Fig. 9B). Furthermore, qPCR results demonstrated that the expression level of MIA PaCa-2 is relatively higher and Patu8988 is lower in EMSLR than other cell lines. Thus, MIA PaCa-2 and Patu8988 were respectively considered as reasonable representatives of the high- and low-risk groups. In order to find effective immunotherapeutic drugs to inhibit the proliferation of PDAC, Phenformin and Pyrimethamine were selected as highly sensitive drugs targeting the high- and the low- risk groups, respectively.Then, HPDE, MIA PaCa-2 and Patu8988 were respectively treated with Phenformin and Pyrimethamine by a defined concentration gradient (0, 1, 10, 50, 100, 200, 500, 1000µM) for 36 h. The drug sensitivities of Phenformin and Pyrimethamine for each the cell line were obtained by calculating their values of IC50 (1041µM for HPDE in Phenformin, 364.8µM for MIA PaCa-2 in Phenformin, 796.9µM for Patu8988 in Phenformin, 461.7µM for HPDE in Pyrimethamine, 336.2µM for MIA PaCa-2 in Pyrimethamine, 193.9µM for Patu8988 in Pyrimethamine), displaying that MIA PaCa-2 was more sensitive for Phenformin than Patu8988 and HPDE and Patu8988 had stronger sensitivity for Pyrimethamine compared with MIA PaCa-2 and HPDE (Fig. 10A, B). Next, the inhibition of MIA PaCa-2 treated with Phenformin was gradually increasing in a dose-dependent manner by CCK8 assay (Fig. 10C). Intriguingly, CCK8 assay revealed that Pyrimethamine facilitated the proliferation of Patu8988 at low concentration. However, the proliferation of Patu8988 was obviously inhibited when it was intervened using Pyrimethamine at a relatively high dose (Fig. 10D). Collectively, appropriate immunotherapy drugs effectively inhibit the proliferation of PDAC, demonstrating their potential as therapeutic agents.

## Discussion

In order to explore the role of lncRNAs and m6A in the development of PDAC, m6A-related lncRNAs were screened by matching m6A and lncRNAs using co-expression analysis. The patients with PDAC were classified into the high- and low-risk groups by univariate Cox analysis and Lasso regression model. The survival of patients with PDAC was excellently predicted by ROC curves of the clinical parameters and risk scores in the risk prognosis model.

As a kind of commonly chemical modification in eukaryotic mRNAs and lncRNAs, abnormal expression of m6A influence cell self-renewal, differentiation, apoptosis, invasion [26, 27]. Zhang H et al. found that the highly expressed SNHG17 modified by METTL3 aggravated the malignant phenotypes in Lung adenocarcinoma (LUAD) gefitinib resistance cells [28]. Li B et al. demonstrated that lncRNA WEE2-AS post-transcriptionally stabilized by IGF2BP3 promotes Glioblastoma (GBM) progression [29]. However, little research focused on pathogenic mechanism of lncRNAs and m6A in PDAC were identified. Therefore, it is essential to explore the prognostic signature about m6A-related lncRNAs in the PDAC.

In our research, 4 m6A-related lncRNAs (including EMSLR, AL358944.1, ZNF236-DT, AC087501.4) were selected to construct a prognosis model. It is reported that the highly expressed EMSLR facilitates proliferation of lung cancer by interacting with target gene LncPRESS [30]. AL358944.1 is a prognostic indicator in the PDAC in the cuproptosis-related lncRNAs signature [31]. Moreover, the numerous evidences showed that tumor cells not only accelerate growth but also inhibit TMB by interacting the infiltered immune cell around tumor cells [32]. GO analysis revealed that immune function between the high- and the low-risk groups were widely disparate. For example, Type-I-IFN Response, MHC-I, T cell receptor complex (TCR) and so on. IFNs, a kind of cytokines, affect the growth, migration and presentation of neonatal antigens. cytotoxic lymphocyte can be stimulated by IFNs, thus, the disturbed IFN responses leading to the loss of tumor immune surveillance [33]. Cytotoxic T lymphocytes can recognize and bind the antigenic peptide-MHC-I complex through TCR receptors to initiate anti-tumor immune function. However, the downregulation of MHC-I leads to the inability of CD8 + T cells to recognize tumor antigen peptides [34], which is closely related to the immunosuppression and poor prognosis of many malignant tumors. Yamamoto K et al. demonstrated that MHC-I is degraded by binding with NBR1 in the autophagy process, promoting immune escape from pancreatic cancer [35].

TIDE (Tumor Immune Dysfunction and Exclusion) is a computational method used to assess the efficacy of immune checkpoint blockade therapy. In general, a lower TIDE score indicates a higher possibility for the response to immunotherapy. In our study, lower TIDE scores were obtained in patients from the high-risk groups compared to those from the low-risk groups, suggesting that those patients with lower TIDE scores may have reduced immune dysfunction and exclusion, a better response to immunotherapy. In the future research, a novel treatment strategy may be found by the research based on TIDE scores, and more options are offered for personalized treatment.

Besides, 12 immunotherapy drugs about PDAC from GDSC websites were filtered, which paves the way for patients with PDAC in medical treatments. Pyrimethamine (2,4-diamino-5-p-chlorophenyl-6-ethylpyrimidine), a dihydrofolate reductase (DHFR) inhibitor, is used for treating malaria by inhibiting the proliferation of plasmodium and toxoplasma and also applied to anti-tumor field [36]. Furthermore, Phenformin, a common biguanide drug, can effectively treat diabetes. Currently, it has been widely used to resist the development of cancers [37]. The proliferation of MIA PaCa-2 treated with Phenformin and Patu8988 treated with Pyrimethamine was inhibited, indicating Phenformin and Pyrimethamine may be potential highly sensitive immunotherapy drugs. In this way, patients with PDAC in the high- and low-risk groups are more likely to be effectively treated when they receive suitable immunotherapy drugs. The established m6A-related lncRNAs model provides a new strategy for clinical outcome in PDAC.

Nevertheless, there are some limitations to our study that need to be addressed. Firstly, although we validated the risk model using separate training and test cohorts, further validation with larger patient cohorts is needed to confirm its reliability and generalizability. Secondly, the underlying molecular mechanisms linking the identified m6A-related lncRNAs to PDAC pathogenesis and progression remain largely unknown. Future studies should focus on elucidating these mechanisms to gain a deeper understanding of the role of m6A-related lncRNAs in PDAC. Lastly, while our study identified potential immunotherapy drugs based on the risk model, further preclinical and clinical investigations are required to evaluate their efficacy and safety in PDAC patients.

## Conclusions

In a word, we successfully constructed and validated a promising risk prognosis model about m6A-related lncRNAs in PDAC. Furthermore, we filtered highly sensitive immunotherapeutic drugs according to the high- and the low-risk groups and validated them in vitro. Our findings are expected to provide effective biomarkers, immunotherapy drugs and a novel method for the diagnosis and treatment of pancreatic cancer. The research laid a solid foundation for further exploration of pathological mechanism and clinical therapeutic schedule.

### Electronic supplementary material

Below is the link to the electronic supplementary material.

**Supplementary Material 1: Figure S1.** Kaplan-Meier curves of OS differences in high- and low-risk groups. **Figure S2.** Prognostic validation of m6A-related lncRNAs in risk model in the TCGA entire sets. **Figure S3.** Prognostic validation of m6A-related lncRNAs in risk model in the test sets. **Figure S4.** Kaplan-Meier curves of OS differences layered by stage I-II (A), III-IV (B), M0 (C), M1 (D) between the high- and low-risk groups from TCGA data set. **Supplementary Table 1.** Primers applied to qPCR analyses

## Data Availability

Publicly available datasets analyzed in this study are available in the Cancer Genome Atlas (Repository (cancer.gov)), UCSC Xena database (http://xena.ucsc.edu), GENCODE website (https://www.gencodegenes.org), SRAMP database (http://www.cuilab.cn/sramp/), GDSC(https://www.cancerrxgene.org/).

## Abbreviations

PDAC: Pancreatic adenocarcinoma
m6A: N6-methyladenosine
lncRNAs: long non-coding RNAs
LASSO: the least absolute shrinkage and selection operator
DEGs: the differential genes
OS: overall survival
SKCM: skin cutaneous melanoma
HPDE: human normal pancreatic duct epithelial cell
IC50: the half-maximal inhibitory concentration
TIDE: tumor immune dysfunction and exclusion
TMB: tumor mutation burden
CI: confidence interval
AUC: the area under the ROC curve
ICIs: Immune checkpoint inhibitors
DHFR: dihydrofolate reductase
GO: Gene Ontology
PCA: Principal component analysis
