# Supplementary figures and images for: Identification of a novel m6A-related lncRNAs signature and immunotherapeutic drug sensitivity in pancreatic adenocarcinoma
Source: BMC Cancer. 2024 Jan 23;24:116. doi: 10.1186/s12885-024-11885-8 (PMC10804632; doi:10.1186/s12885-024-11885-8)

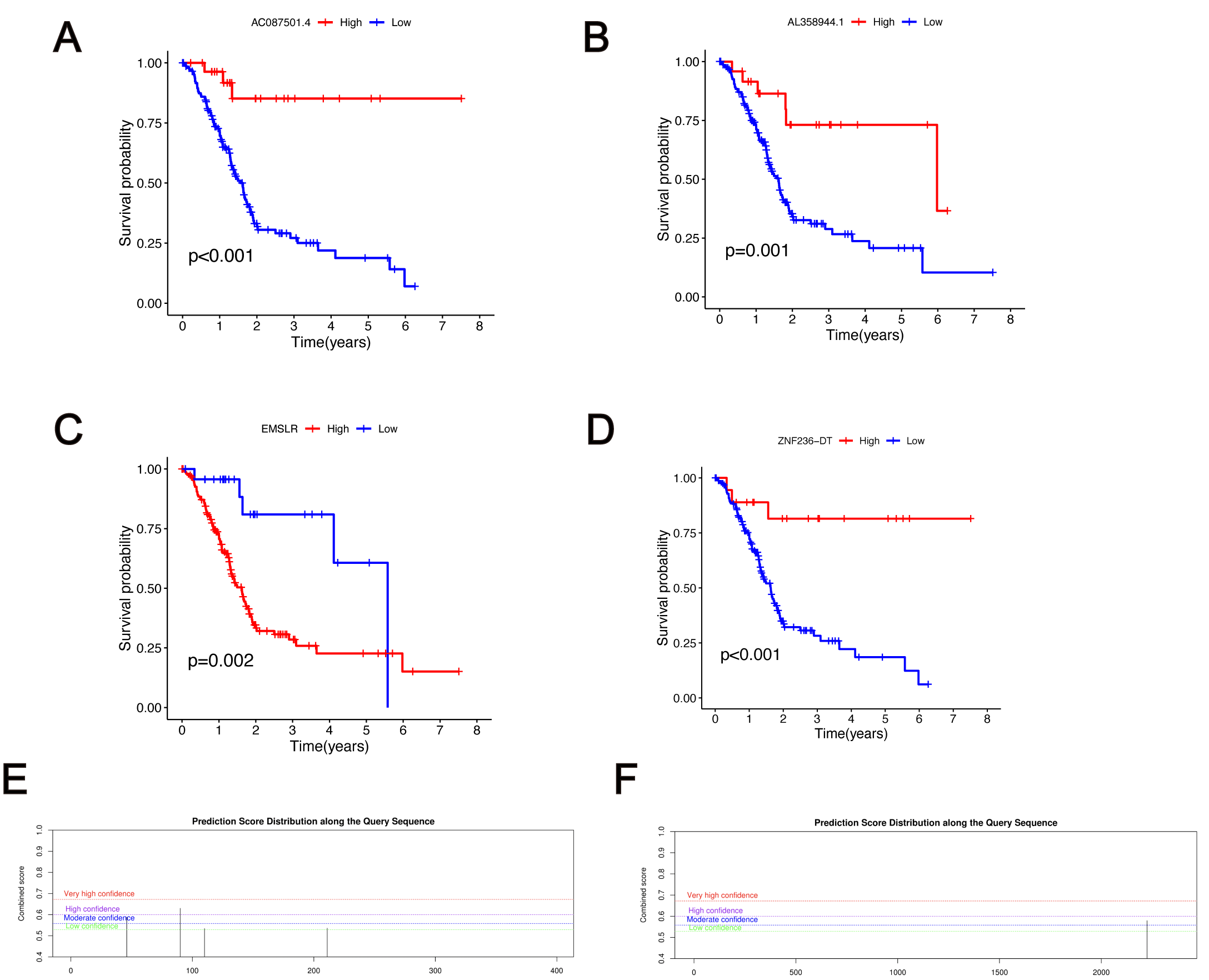

Supplement: Supplementary file 1 — Supplementary Material 1: Figure S1. Kaplan-Meier curves of OS differences in high- and low-risk groups. Figure S2. Prognostic validation of m6A-related lncRNAs in risk model in the TCGA entire sets. Figure S3. Prognostic validation of m6A-related lncRNAs in risk model in the test sets. Figure S4. Kaplan-Meier curves of OS differences layered by stage I-II (A), III-IV (B), M0 (C), M1 (D) between the high- and low-risk groups from TCGA data set. Supplementary Table 1. Primers applied to qPCR analyses [file 12885_2024_11885_MOESM1_ESM.zip › Supplementary Material2/FigureS1.tif]

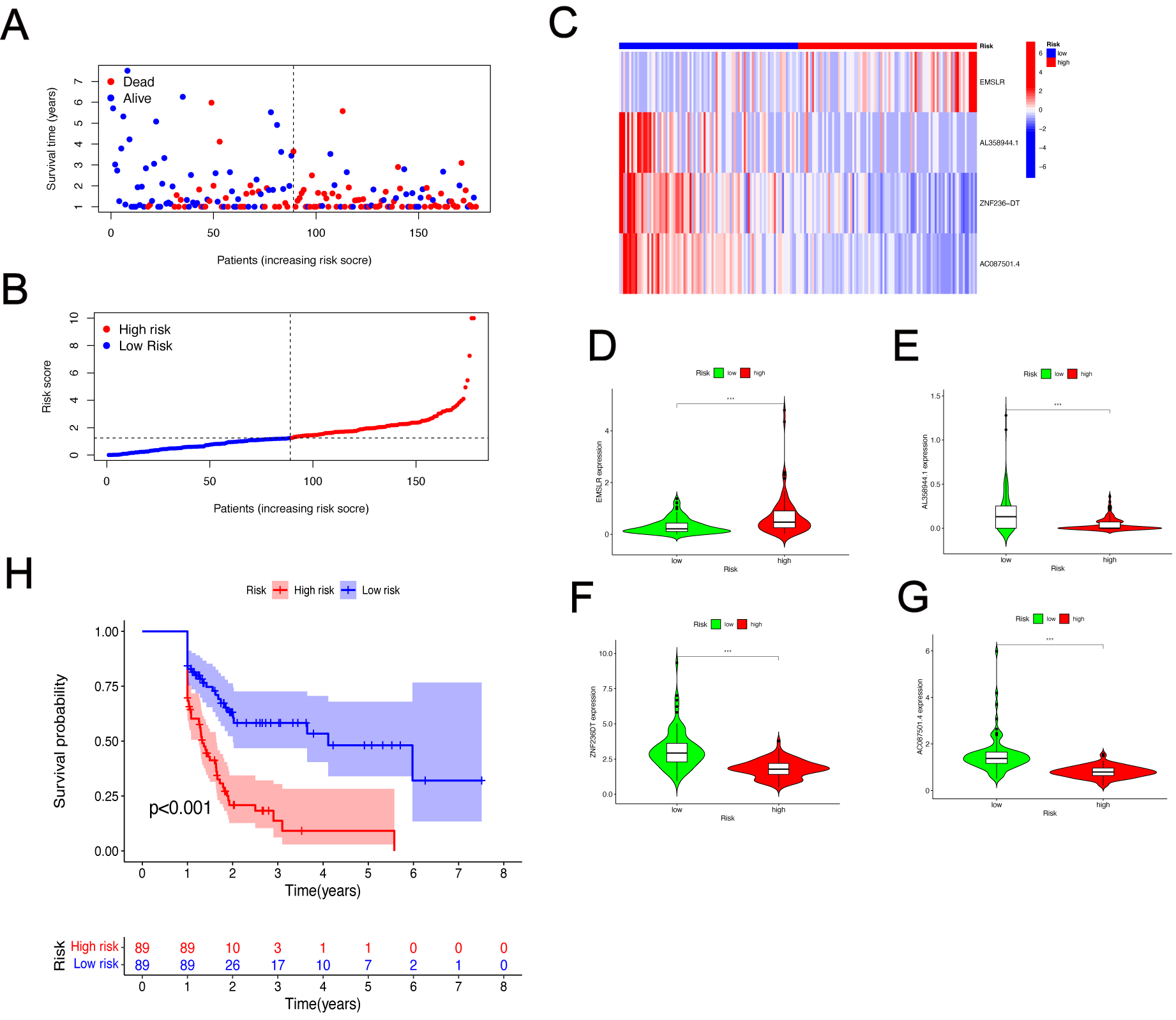

Supplement: Supplementary file 1 — Supplementary Material 1: Figure S1. Kaplan-Meier curves of OS differences in high- and low-risk groups. Figure S2. Prognostic validation of m6A-related lncRNAs in risk model in the TCGA entire sets. Figure S3. Prognostic validation of m6A-related lncRNAs in risk model in the test sets. Figure S4. Kaplan-Meier curves of OS differences layered by stage I-II (A), III-IV (B), M0 (C), M1 (D) between the high- and low-risk groups from TCGA data set. Supplementary Table 1. Primers applied to qPCR analyses [file 12885_2024_11885_MOESM1_ESM.zip › Supplementary Material2/FigureS2.tif]

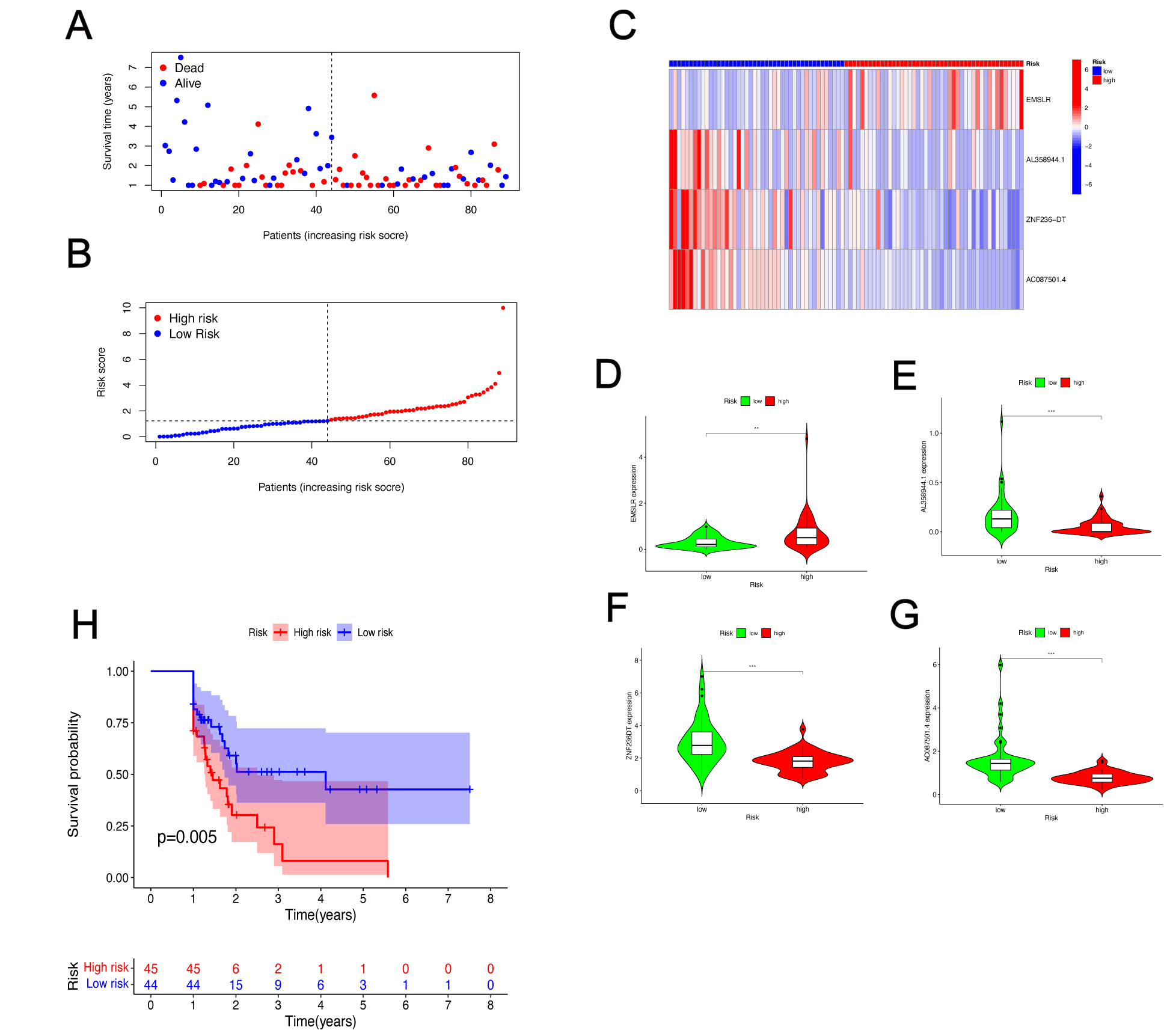

Supplement: Supplementary file 1 — Supplementary Material 1: Figure S1. Kaplan-Meier curves of OS differences in high- and low-risk groups. Figure S2. Prognostic validation of m6A-related lncRNAs in risk model in the TCGA entire sets. Figure S3. Prognostic validation of m6A-related lncRNAs in risk model in the test sets. Figure S4. Kaplan-Meier curves of OS differences layered by stage I-II (A), III-IV (B), M0 (C), M1 (D) between the high- and low-risk groups from TCGA data set. Supplementary Table 1. Primers applied to qPCR analyses [file 12885_2024_11885_MOESM1_ESM.zip › Supplementary Material2/FigureS3.tif]

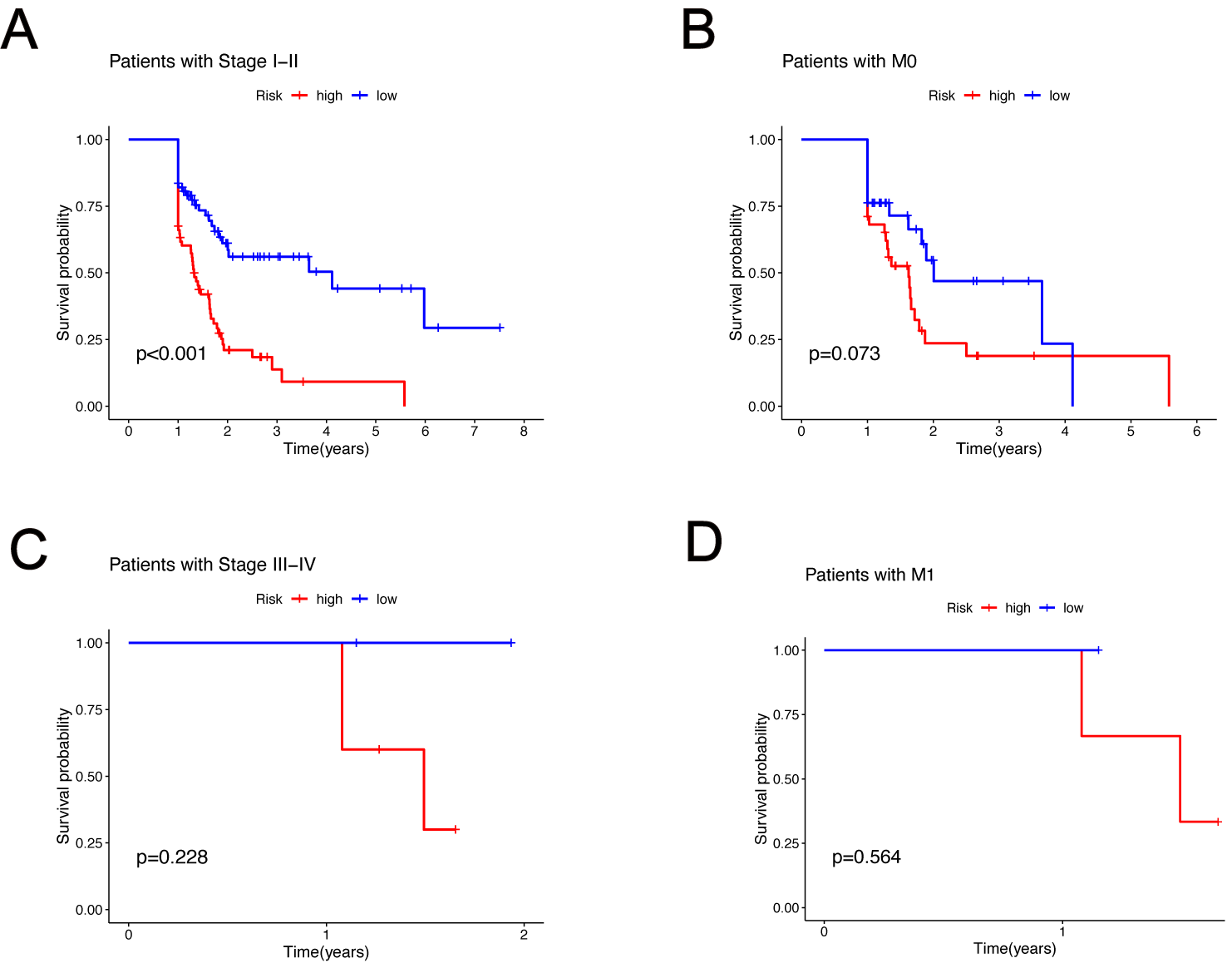

Supplement: Supplementary file 1 — Supplementary Material 1: Figure S1. Kaplan-Meier curves of OS differences in high- and low-risk groups. Figure S2. Prognostic validation of m6A-related lncRNAs in risk model in the TCGA entire sets. Figure S3. Prognostic validation of m6A-related lncRNAs in risk model in the test sets. Figure S4. Kaplan-Meier curves of OS differences layered by stage I-II (A), III-IV (B), M0 (C), M1 (D) between the high- and low-risk groups from TCGA data set. Supplementary Table 1. Primers applied to qPCR analyses [file 12885_2024_11885_MOESM1_ESM.zip › Supplementary Material2/FigureS4.tif]
